# Involvement of Phosphatase and Tensin Homolog in Cyclin-Dependent Kinase 4/6 Inhibitor-Induced Blockade of Glioblastoma

**DOI:** 10.3389/fphar.2019.01316

**Published:** 2019-11-07

**Authors:** Songlin Liu, Dun Yuan, Yifeng Li, Qi Qi, Bingzhong Guo, Shun Yang, Jilin Zhou, Lu Xu, Tiange Chen, Chenxing Yang, Junyu Liu, Buyan Li, Li Yao, Weixi Jiang

**Affiliations:** ^1^Department of Neurosurgery, Xiangya Hospital, Central South University, Changsha, China; ^2^Department of Pharmacology, Clinical Translational Center for Targeted Drug, School of Medicine, Jinan University, Guangzhou, China

**Keywords:** glioblastoma, PTEN, CDK4/6 inhibitor, palbociclib, sensitivity

## Abstract

Dysregulation of retinoblastoma (Rb) signaling pathway have been established as a requirement for glioblastoma (GBM) initiation and progression, which suggests that blockade of CDK4/6-Rb signaling axis for GBM treatment. Palbociclib, a selective inhibitor of the cyclin-dependent kinases CDK4/6, has been applied for breast cancer treatment. However, its efficacy against glioblastoma has not been well clarified. Here, effects of CDK4/6 inhibitors on various kinds of GBM cell lines are investigated and the functional mechanisms are identified. Data showed that cells with diverse PTEN status respond to palbociclib differently. Gain-of-function and loss-of-function studies indicated that PTEN enhanced the sensitivity of GBM cells to palbociclib *in vitro* and *in vivo*, which was associated with suppressions of Akt and ERK signaling and independent of Rb signaling inhibition. Hence, our findings support that palbociclib selectively

## Introduction

Gliomas constitute about 30% of brain tumors and central nervous system tumors in adults and grade IV glioma, glioblastoma (GBM), ranks among the highest malignant types of human cancer due to its dismal prognosis (Jing et al., 2017; Qiu et al., 2017). Although combination of surgery, radiotherapy, and chemotherapy are employed for treating GBM (Huang et al., 2017), the mean patient survival time reaches only 14.6 months (Wilson et al., 2014) and the 5-year survival rate is merely 5.6% (Patel et al., 2019). Therefore, it is urgent to identify alternative therapeutic approaches, as well as to explore the comprehensive molecular mechanisms underlying GBM initiation and progression. The Cancer Genome Atlas Research Network (TCGA) has demonstrated that dysfunctions of receptor tyrosine kinase (RTK), PI3-kinase, and Rb signaling are the three key pathways contributing to GBM initiation and progression (Brennan et al., 2013). Among them, Rb signaling pathway is the most promising target for clinical application since inhibitors of Rb signaling has not been well developed for GBM treatment (Cen et al., 2012; Schroder and McDonald, 2015).

**Figure 1:**
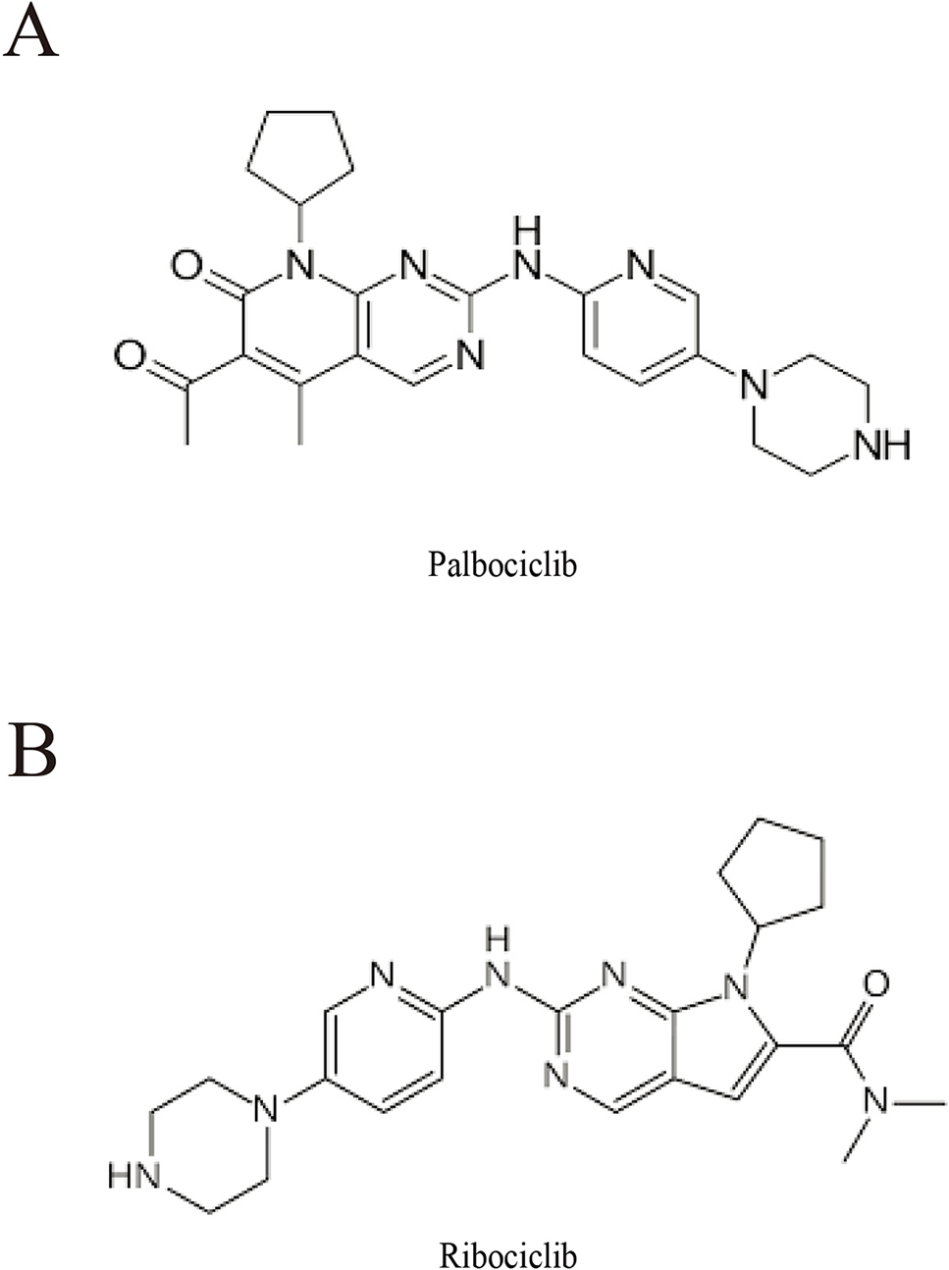
Chemical structure of palbociclib **(A)** and ribociclib **(B)**.

The cyclin dependent kinase (CDK) 4/6 controls cell cycle progression *via* modulating the G1/S checkpoint. CDK4/6 mediates the process from G1 to S phase by interaction with D-type cyclins and regulating Rb phosphorylation. Upregulated cyclin D/CDK4/6 activity induces Rb phosphorylation and ultimately leads to tumor growth (Chen and Pan, 2017). Selective CDK4/6 inhibitors provide a novel therapeutic approach for patients with malignant tumors. Palbociclib (**Figure 1A**) and ribociclib (**Figure 1B**) are the most well characterized CDK4/6 inhibitors and abemaciclib, the third CDK4/6 inhibitor has been approved for clinical use most recently (Tate et al., 2018). Palbociclib, also named PD-0332991, is the first CDK4/6 inhibitor approved for cancer therapy (Finn et al., 2015; Hamilton and Infante, 2016). It functions as antitumor agent against hepatocellular carcinoma (Bollard et al., 2016), synovial sarcoma (Vlenterie et al., 2016), head and neck squamous cell carcinoma (Michel et al., 2016), liposarcoma (Dickson et al., 2013), non-small cell lung cancers (Tao et al., 2016), as well as GBM (Whittaker et al., 2017; Liu et al., 2018). Palbociclib can pass through the blood–brain barrier (Michaud et al., 2010; de Gooijer et al., 2015) and has been approved for phase II clinical study for GBM treatment (Schroder and McDonald, 2015; Taylor et al., 2018). However, mechanisms by which CDK4/6 inhibitor suppresses GBM progression and its selectivity against GBM with various genetic backgrounds still need to be defined.

The PI3K/Akt pathway is a downstream pathway of RTK signaling, which contributes to GBM as one of the three key signaling pathways. Phosphatase and tensin homolog (PTEN), a phosphatase, catalyses dephosphorylation of the 3′-phosphate of the inositol ring in phosphatidylinositol 3,4,5-trisphosphate (PIP3), changing to the biphosphate product phosphatidylinositol 4,5-bisphosphate (PIP2), resulting in inhibition of the Akt signaling pathway. Located on chromosome 10q23.3, PTEN is one of the highest deficient or mutated genes in brain, breast cancer, and prostate tumors. As a tumor suppressor, its production implicates in various cellular processes such as metabolism, apoptosis, and cell proliferation through blocking PI3K/Akt pathway (Luo et al., 2018). It also plays important roles in both neurogenesis and gliogenesis (Chenn and Walsh, 2002; Fraser et al., 2004) and mutations of PTEN are involved in the malignant progression of glioma (Rasheed et al., 1997). Therefore, PTEN is a promising molecular marker and a prognosis marker for GBM treatment.

In the present study, we investigated the effects of CDK4/6 inhibitor on various kinds of GBM cell lines and find out that cells with diverse PTEN status respond to palbociclib differently. Further studies demonstrated that PTEN expression sensitized GBM cells to palbociclib both *in vitro* and *in vivo*, which was involved in selective blockade of Akt and ERK signaling. Data also indicated that the suppression of Akt and ERK signaling by palbociclib was independent of suppression of Rb signaling. Hence, our findings support that palbociclib selectively suppresses GBM with wild-type PTEN, providing preclinical evidence and a proof-of-concept that CDK4/6 inhibitors are promising drugs for GBM treatment.

## Materials and Methods

### Chemical and Reagents

Palbociclib and ribociclib were from Elleckchem. They were prepared to 10 mM with dimethyl sulfoxide (DMSO) as stock solution and stored at −20°C. Then they were freshly diluted with cell culture medium to certain concentrations. 3-(4,5-dimethylthiazol-2-yl)-2, 5-diphenyltetrazol-iumbromide (MTT), antibody against β-actin (Cat# A2228), and monoclonal anti-HA-peroxidase antibody (Cat# H6533) were purchased from Sigma. Anti-Myc-peroxidase antibody (Cat# R951-25) was from Thermo Fisher. BrdU incorporation assay kit (#6831) and antibodies against PTEN (Cat# 9188), phospho-Rb (Ser280) (Cat# 8181), Rb (Cat# 9309), phospho-Akt (Ser473) (Cat# 4060), Akt (Cat# 4685), phospho-ERK (Thr202/Tyr204) (Cat# 4370), ERK (Cat# 4695), phospho-GSK-3β (Ser9) (Cat# 5558), GSK-3β (Cat# 12456), phospho-Elk-1 (Ser383) (Cat# 9186), and Elk-1 (Cat# 9182) were from Cell signaling (Beverly, MA). The horseradish peroxidase linked IgG secondary antibodies were obtained from GE Healthcare. Plasmids of constitutively active Akt (CA, Cat# 14751) (Scheid et al., 2002), ERK (CA, Cat# 39194) (Robinson et al., 1998), and PTEN (#78776) were from Addgene. PTEN siRNA (Cat# sc-29459) and control siRNA (Cat# sc-36369) were purchased from Santa Cruz. All chemicals and reagents not described above were from Sigma-Aldrich (St. Louis, MO).

### Cell Lines and Cell Culture

Five human GBM cell lines: U87MG, LN229, LNZ308, SF763, and U251 were from the American Type Culture Collection (ATCC, Manassas, VA, USA). Among them, LN229 and SF763 are with wild-type PTEN (Liu et al., 2018). U87MG, U251, and LNZ308 cells are with mutated/deficient PTEN (Levitt et al., 2005; Liu et al., 2018). The cells were cultured in DMEM medium (Gibco, Grand Island, NY, USA) supplemented with 10% fetal bovine serum. PTEN stable transfected U87MG cells (U87MG-PTEN) and vector control cells (U87MG-Vector) were cultured with the completed medium described above supplemented with G418 (400 µg/ml) (Qi et al., 2012). All cells were maintained in a 37°C, 5% CO_2_ humidified incubator.

### Cell Proliferation and Colony Formation Assay

Before treatment, cells were seeded in 96-well plates (3,000 cells/well). The next day, medium of the cells were replaced with completed medium with various concentrations of test drugs. Experiments were performed three times in a parallel manner. After treatment, 0.5 mg/ml MTT solution was added to each well. The plate was further maintained at 37°C for 4 h. Then 100 µl of DMSO was added to each well followed by discarding the supernatant. The mixture was dissolved and measured at 570 nm. For colony formation assay, cells were seeded (5,000 cells/well) with 0.4% agar gel dissolved in DMEM medium with 10% FBS in a well (six-well plate) with a base coated with 0.6% agar gel. Fresh completed medium with drug/vehicle was changed every 2 days. Following treatment for 3 weeks, colonies were stained with MTT and quantified.

### Plasmids and siRNA Transfection

Cells with 60∼70% confluent density were transfected with certain plasmids or siRNA using Lipofectamine 3000 (Invitrogen, Carlsbad, CA) according to the data sheet of the product. Twelve hours after transfection, fresh completed medium was added and cells were maintained for another 16 h before examinations.

### Tumor Xenograft Model

Female athymic BALB/c nude mice (35–40 days old, 18–22 g) were purchased from Shanghai Institute of Materia Medica, Chinese Academy of Sciences. U87MG-PTEN/Vector cells (4 × 10^6^) in 100 µl of serum-free DMEM medium were inoculated subcutaneously into one flank of the mouse. Procedures were approved by the Committee on the Ethics of Animal Experiments of Jinan University. Drug administration was started when tumors reached a mean volume of 100 mm^3^. For each model, the mice were randomly grouped and treated with vehicle (50 mM sodium lactate) and palbociclib (150 mg/kg/day) orally for 3 weeks (Michaud et al., 2010). Tumor volume (mm^3^) was calculated using the formula: (length × width^2^)/2. Data were expressed as mean tumor volume ± SD of each group. The animals were sacrificed and samples/data were collected followed 24 h after the last treatment.

### Reverse Transcription-Polymerase Chain Reaction (RT-PCR) and Western Blot Assay

Total RNA was extracted with RNA isolation kit from Zymo. RT-PCR reaction system was constructed according to the protocol supplied with TaKaRa kit. Glyceraldehyde-3-phosphate dehydrogenase (GAPDH) was used as an internal control. The primers were synthesized by Sanggon as follows: PTEN (5′–3′), forward-TGA CAG CCA TCA TCA AAG AG, reverse-TGT GTA TGC TGA TCT TCA TCA A; GAPDH (5′–3′), forward-AAC GTG TCA GTO GTG GAC CT, reverse-AGT GGG TGT CGC TGT FGA AGT. The amplified PCR products were identified by electrophoresis in a 1.5% agarose gel. Western blot assay was carried out as previously described (Qi et al., 2013b).

### Statistical Analysis

Quantitative data are presented as the mean ± SD of three independent experiments. Statistical comparisons were evaluated by the Student’s t-test or one-way ANOVA. A value of *P* < 0.05 was considered statistically significant. Statistical analysis were performed using Prism software (GraphPad Software, La Jolla, CA, USA).

## Results

### Human GBM Cells With Wild-Type PTEN Are More Sensitive to CDK4/6 Inhibitor

**Figure 2:**
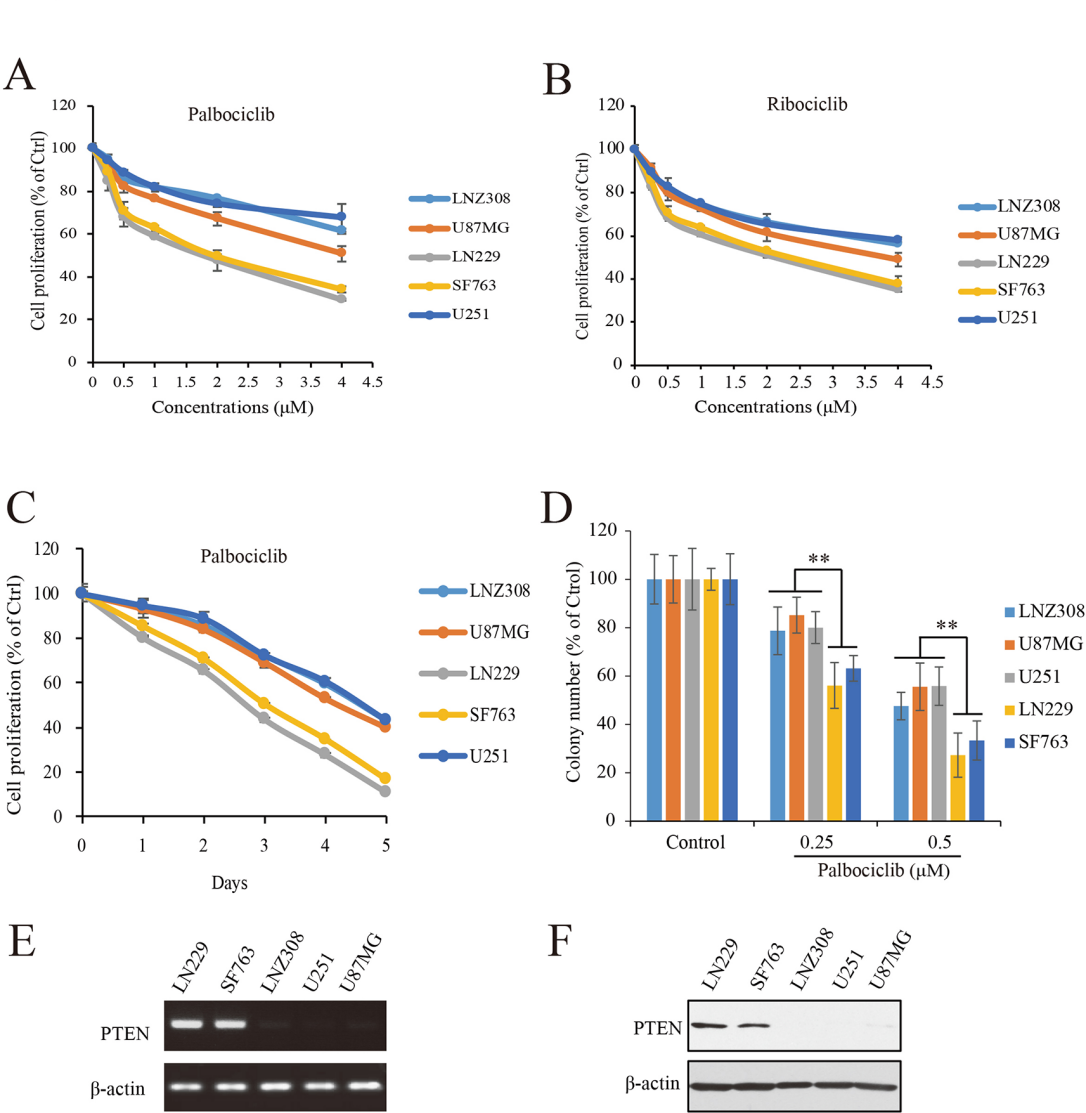
Effects of CDK4/6 inhibitor on various GBM cells with different PTEN status. **(A**, **B)**. Cells were seeded in 96-well plate (3,000 cells/well) and treated with indicated concentrations of palbociclib **(A)** and ribociclib **(B)** for 48 h. Cell proliferation was assessed by MTT assay as described in *Materials and Methods*. Data are expressed as mean ± SD. **(C)** Palbociclib suppressed GBM cells in a time-dependent manner. Cells were prepared as described above and treated with palbociclib (0.5 μM) for indicated times. Cell proliferation was assessed by MTT assay as described in *Materials and Methods*. Data are expressed as mean ± SD. **(D)** Effects of palbociclib on colony formation in GBM cells with different PTEN status. Data are show as means ± SD (**P 0.01, *t*-test, *n* = 3). **(E)** PTEN expression profile at mRNA level in the tested GBM cell lines. **(F)** PTEN expression profile at protein level in the tested GBM cell lines.

To study the efficacy of CDK4/6 inhibitor against human GBM, actions of palbociclib and ribociclib we firstly examined with five human GBM cell lines: NZ308, U87MG, LN229, SF763, and U251. Following treatments with different concentrations of palbociclib for 48 h, the cells were subjected to MTT assay for proliferation examination. As shown in **Figure 2A**, cell proliferation of all the cells was inhibited by palbociclib in a concentration-dependent manner. Interestingly, the sensitivities of these cell lines to palbociclib were distinct. Among them, wild-type PTEN expressed LN299 and SF763 cells were more sensitive to palbociclib, with IC50 values of 1.89 and 2.07 µM, respectively. To confirm the observed phenomenon in GBM cells treated with palbociclib, another CDK4/6 inhibitor, ribociclib was employed. Following treatment for 48 h, although the inhibitive effects of ribociclib in GBM cells were weaker than those of palbociclib, LN299 and SF763 cells were also the most sensitive cells to ribociclib (**Figure 2B**). Further time-course test was carried out to examine the effects of palbociclib in these GBM cells. Data showed that palbociclib inhibited GBM cells in a time-dependent manner and palbociclib selectively suppressed LN299 and SF763 cells (**Figure 2C**). To confirm the selectivity of palbociclib among these GBM cell lines, colony formation assay was conducted. Data showed that palbociclib selectively suppression the colony formation of LN229 and SF763 cells compared to other cells (**Figure 2D**, **Supplemental Figure 1**). The expressions of PTEN at both mRNA and protein levels were confirmed (**Figures 2E, F**). Collectively, these data indicate that GBM cells with wild-type PTEN are more sensitive to CDK4/6 inhibitor, suggesting a role of PTEN in the selectivity of palbociclib against GBM.

### Identification of the Role of PTEN in the Inhibition of GBM Cells Induced by Palbociclib

**Figure 3:**
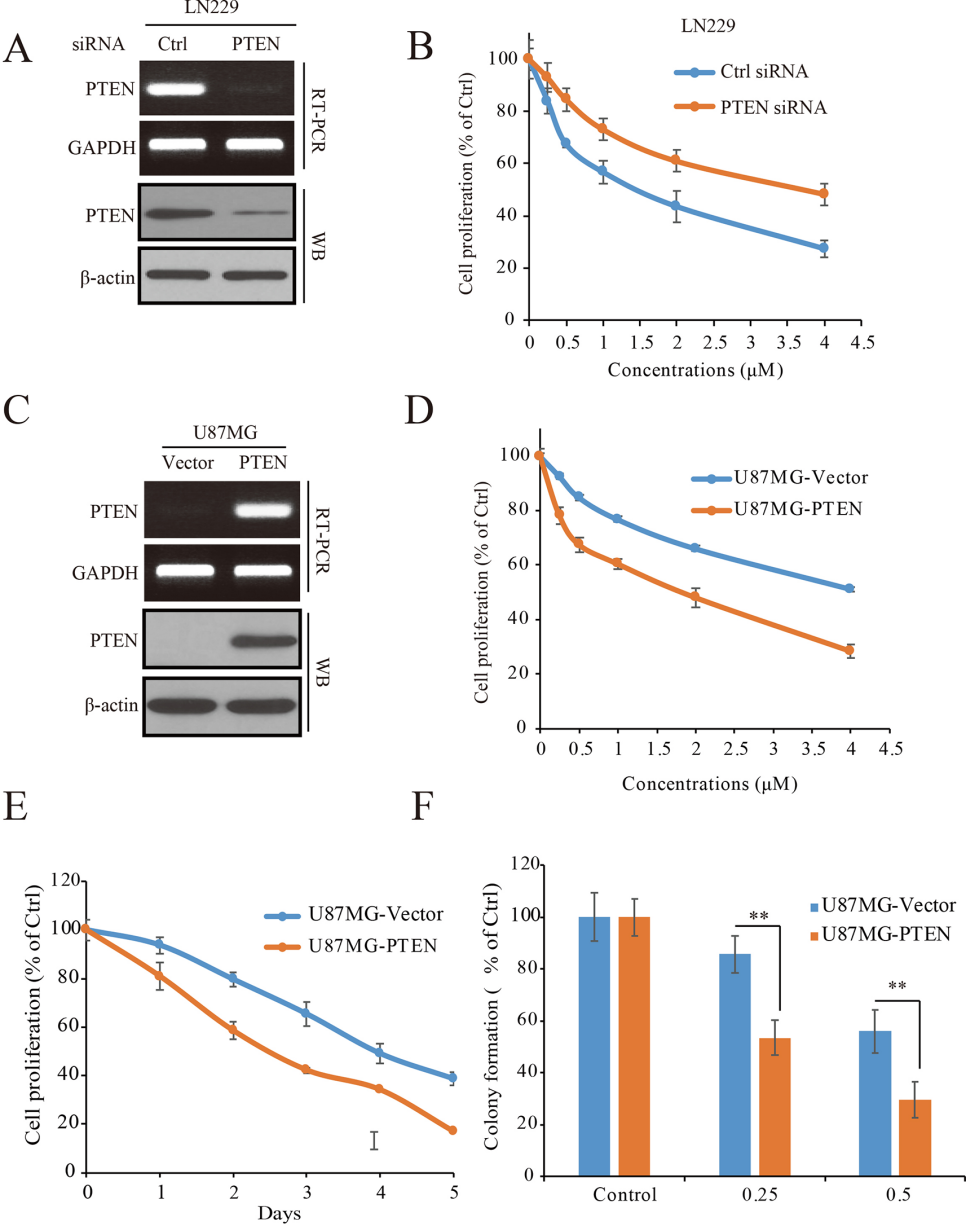
PTEN enhances the sensitivity of GBM cells to palbociclib. **(A)** Detection of PTEN expression at mRNA and protein levels in cells transfected with PTEN/control siRNA. Cell lysates/total RNA were collected and analyzed by western blot/RT-PCR assay as described in *Materials and Methods*. **(B)** PTEN knocking-down decreased the sensitivity of LN229 cells to palbociclib. Following PTEN/control siRNA transfection, cells were treated with palbociclib at indicated concentrations for 48 h. Cell proliferation was examined by MTT assay. Data are expressed as mean ± SD. **(C)** Detection of PTEN expression at mRNA and protein levels in isogenic U87MG cells. Cell lysates/total RNA were collected and examined by western blot/RT-PCR assay. **(D)** Expression of PTEN enhanced the efficacy of palbociclib against U87MG cells. Isogenic U87MG cells with or without PTEN were treated with palbociclib at indicated concentrations for 48 h, cell proliferation was assessed by MTT assay. Data are expressed as mean ± SD. **(F)** Time-course evaluation of palbociclib on isogenic U87MG cells. **(E)** Effects of palbociclib on colony formation of U87MG isogenic cells with different PTEN status. Data are shown as means ± SD (*P 0.05, **P 0.01, *t*-test, *n* = 3).

To determine the role of PTEN in the anti-GBM activity of palbociclib, PTEN expressed LN229 cells were employed to construct a loss-of-function model with siRNA knocking-down operation (**Figure 3A**). Following siRNA treatment, the cells were received palbociclib treatment at various concentrations. Data showed that, compared to control cells, PTEN knocking-down weakened the growth suppression of palbociclib in LN229 cells, with IC50 values increased from 1.78 to 5.66 µM (**Figure 3B**). Similar observations were made in SF763 cells (**Supplemental Figure 2A**). Next, PTEN-null U251 cells were subjected to the proliferation evaluation and found that palbociclib selective suppressed cells transfected with PTEN (**Supplemental Figure 2B**). Furthermore, PTEN stable transfected U87MG isogenic cells were employed for validation (**Figure 3C**). As shown in **Figure 3D**, PTEN expression sensitized cells to palbociclib with IC50 decreased from 4.81 to 2.02 µM. Data from the time-course study also showed that PTEN expression enhanced the sensitivity of U87MG cells to palbociclib (**Figure 3E**). To validate the role of PTEN in palbociclib-induced inhibition of GBM cell, we further employed colony formation assay. Consistent with the results from MTT assay, in the presence with PTEN, palbociclib exerts stronger inhibitive effects on U87MG (**Figure 3F**). Taken together, these data suggest that PTEN contributes to the inhibition of palbociclib in GBM cells and palbociclib selectively suppresses PTEN expressed GBM cells.

### ERK and Akt Signaling Pathways Are Selectively Blocked by Palbociclib in PTEN-Expressed GBM Cells

**Figure 4:**
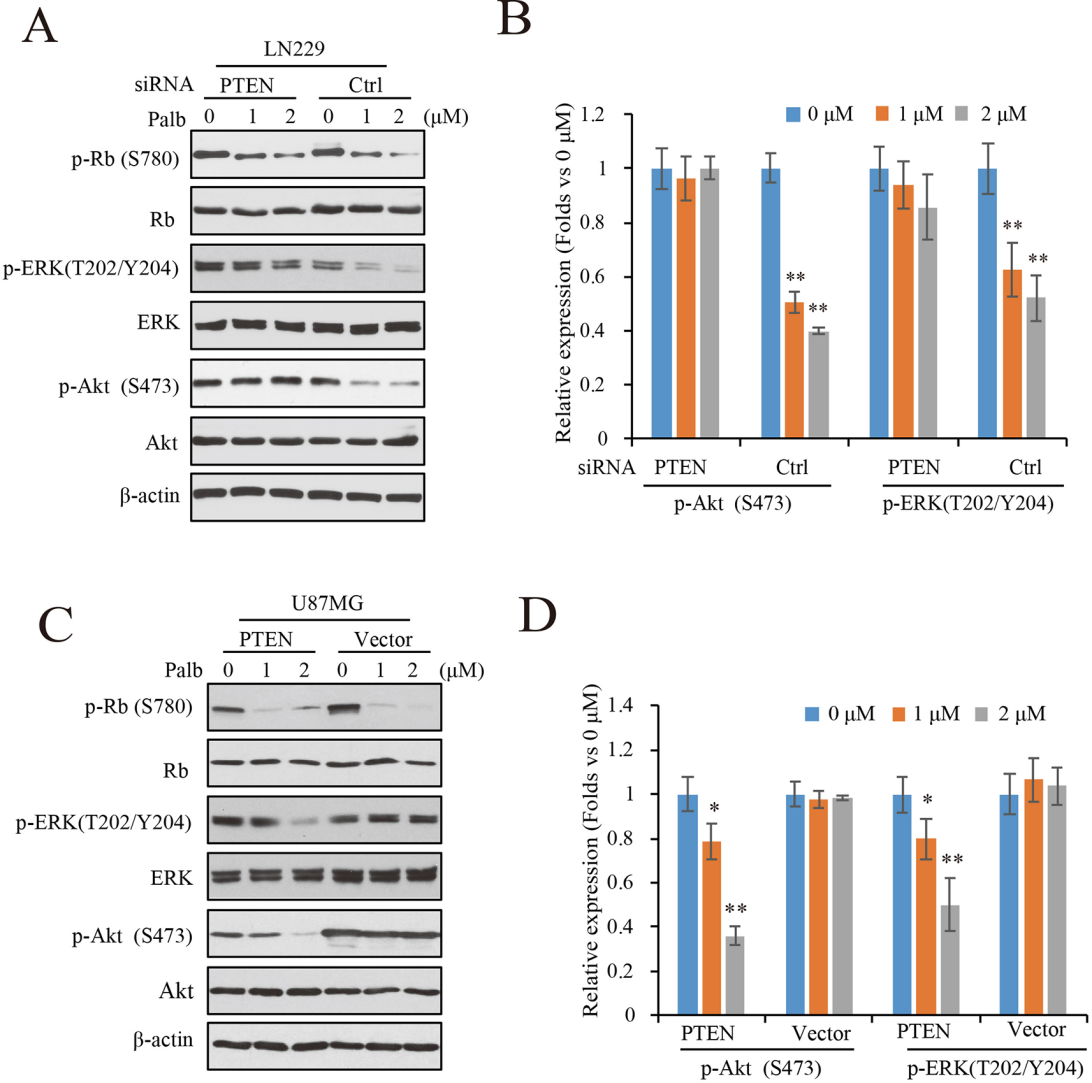
Palbociclib blocks ERK and Akt signaling in the presence of PTEN. **(A)** Effects of palbociclib on the levels of p-ERK, p-Akt, as well as p-Rb in LN229 cells pre-treated by PTEN/control siRNA. Following siRNA treatment, the cells were subjected to palbociclib treatment for 24 h. Cell lysates were collected and analyzed by western blot assay. **(B)** Quantification of the blots in **(A)** Data are shown as mean ± SD (*P 0.05, **P 0.01, one-way ANOVA, *n* = 3). **(C)** Effects of palbociclib on the levels of p-ERK, p-Akt, as well as p-Rb in U87MG isogenic cells. Cells were treated with palbociclib at indicated concentrations for 24 h. Cell lysates were collected and subjected to western blotting analysis with indicated antibodies. **(D)** Quantification of the blots in **(C)**. Data are shown as mean ± SD (*P 0.05, **P 0.01, one-way ANOVA, *n* = 3).

To determine the mechanisms by which PTEN contributes to palbociclib-induced suppression in GBM cells, PTEN knocking-down LN229 cells were treated with palbociclib at various concentrations followed by signaling analysis. Data showed that the levels of Rb phosphorylation were downregulated in both groups with or without PTEN (**Figure 4A**). However, suppressions induced by palbociclib in ERK and Akt signaling were different between PTEN wild-type and PTEN deficient cells. As shown in **Figure 4A**, in LN229 cells with normal PTEN expression, levels of phospho-ERK and phospho-Akt were decreased by palbociclib. However, the effects were abolished in cells pre-treated by PTEN siRNA, showing no differences induced by palbociclib compared to vehicle treated cells (**Figure 4A**). Quantified data showed that palbociclib decreased the levels of phospho-ERK and phospho-Akt significantly in control siRNA treated cells, while no notable suppressions observed in cells treated by PTEN siRNA (**Figure 4B**). Furthermore, the U87MG-Vector/PTEN isogenic cells were employed for validation. Following palbociclib treatment, phospho-Rb levels were reduced in both U87MG isogenic cell lines. However, in PTEN expressed cells, palbociclib decreased the levels of phospho-ERK and phospho-Akt in a concentration-dependent manner, while no distinguished changes was observed in U87MG-Vector cells (**Figure 4C**). Quantification data indicated that, compared to vehicle treated group, palbociclib treatment significantly inhibited the expressions of phospho-ERK and phospho-Akt in U87MG-PTEN cells, while scarcely affected the ERK and Akt signaling in U87MG-Vector cells (**Figure 4D**).

**Figure 5:**
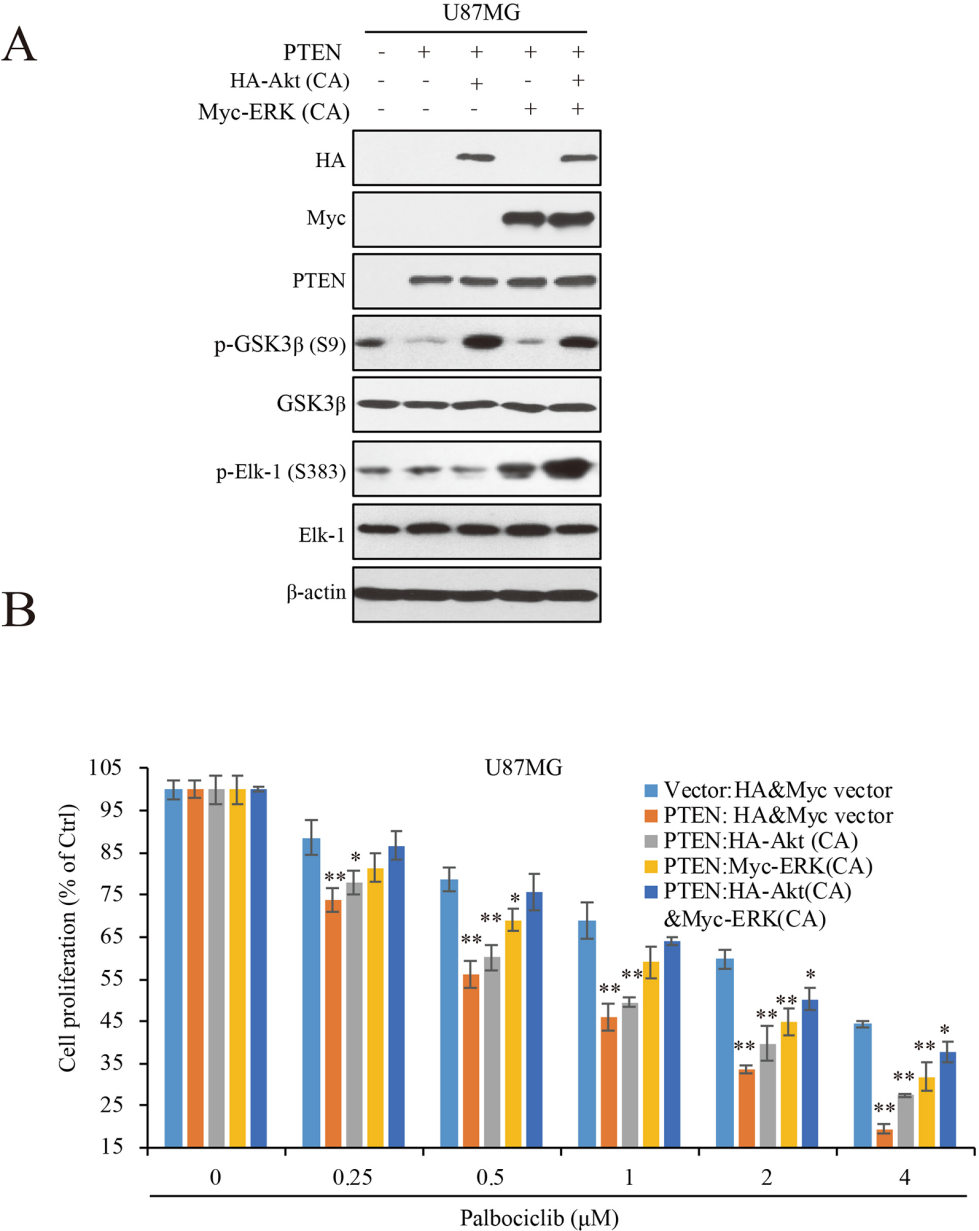
ERK and Akt activation alleviates palbociclib-induced suppression of GBM cells. **(A)** Validation of the cell systems with active ERK or/and Akt activation. U87MG isogenic cells were transfected with ERK-CA or/and Akt-CA plasmids. Cell lysates were collected and examined by western blot assay with indicated antibodies. **(B)** Effects of palbociclib on cell proliferation in the cell systems constructed in **(A)** Following palbociclib treatment, cell proliferation was assessed by MTT assay as described in *Materials and Methods*. Data are expressed as mean ± SD (*P 0.05, **P 0.01, vs. control cells in each dose group, *t*-test, *n* = 3).

To Further Clarify the Roles of ERK and Akt Signaling in PTEN-Induced Sensitivity of GBM Cells to Palbociclib, We Transfected Constitutive Active ERK (ERK-CA) and Akt (Akt-CA) Plasmids Into U87MG-PTEN/Vector Isogenic Cells. Akt Activation Was Enhanced Indicated by Increased Expression of Phospho-GSK3β That Is a Canonical Substrate of Akt (**Figure 5A**). ERK-CA Led to Up-Regulation of Phospho-Elk-1 That Is the Substrate of ERK. in Addition, Akt-CA Additively Enhanced Phosphor-Elk-1 Expression in the ERK-CA Transfected Cells (**Figure 5A**). With the Cell Systems Constructed Above, We Further Examined the Effects of Palbociclib on Cell Proliferation in Cells With High ERK Or/And Akt Activations. Data Showed That, At Low Concentrations (0.25–1 Μm), Co-Transfection of Akt-CA and ERK-CA Alleviated the Suppression Induced by Palbociclib. Compared the Data From Akt-CA With Those From ERK-CA Groups, ERK Activation Exerted Stronger Effect on Eliminating Palbociclib-Induced Inhibitive Effects, Suggesting a Major Role of ERK Signaling in Palbociclib-Induced Blockade of GBM (**Figure 5B**). These Observations Were Also Confirmed by the Brdu Incorporation Assay (**Supplemental Figure 3**). Collectively, These Data Demonstrate That, in the Presence of PTEN, Palbociclib Suppresses GBM Cell Proliferation Through Downregulation of Phospho-ERK and Phospho-Akt Levels, Indicating PTEN Contributes to the Selectivity of Palbociclib Against GBM Through Blocking ERK and Akt Signaling.

### Palbociclib Selectively Blocks the Growth of PTEN-Expressed GBM Cells *In Vivo*

**Figure 6:**
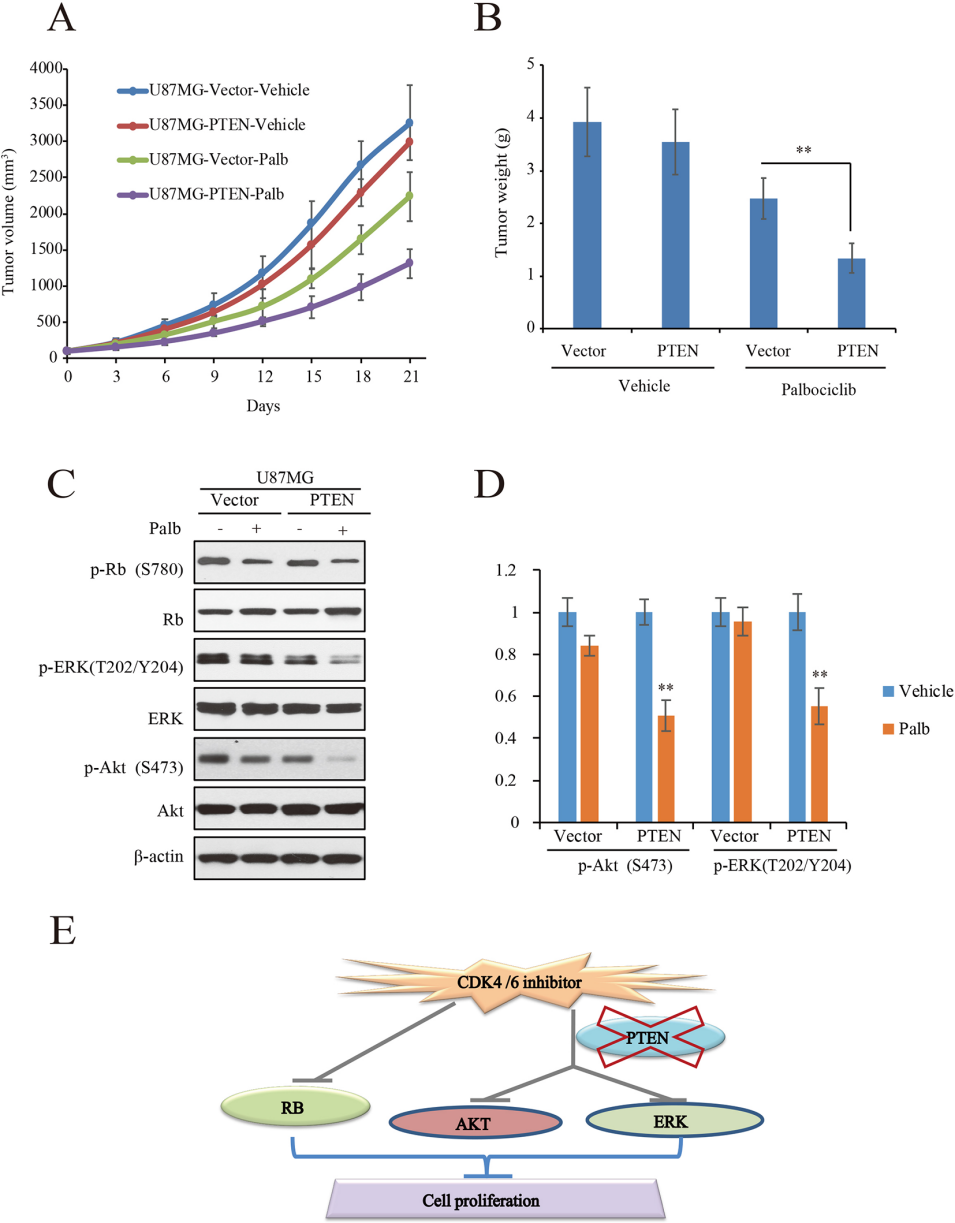
Palbociclib selectively blocks PTEN-expressed U87MG cells *in vivo*. **(A**, **B)** U87MG isogenic cells were inoculated subcutaneously; mice were treated with palbociclib as described in *Materials and Methods*. Tumor volumes **(A)** and tumor weight **(B)** were examined throughout the experiment. Data are shown as mean ± SD (*P 0.05, **P 0.01, *t*-test, eight mice/group). **(C)** Effects of palbociclib on the levels of p-ERK, p-Akt, as well as p-Rb in U87MG-Vector/PTEN tumors. Following treatment, tumor tissues were collected. The lysates were analyzed by western blot assay with indicated antibodies. **(D)** Quantification of the blots in** (C)**. Data are shown as mean ± SD (**P 0.01, vs. vehicle treatment, *t*-test, *n* = 3). **(E)** A schematic diagram

To validate the action of PTEN in palbociclib-induced suppression of GBM cell *in vitro*, *in vivo* tumor xenograft assay was conducted with mouse bearing U87MG-PTEN/Vector isogenic cells. The administration and dosage of palbociclib (150 mg/kg) are proved non-toxic *in vivo* (Michaud et al., 2010). Following treatment with either vehicle or palbociclib daily for 21 consecutive days, tumors in palbociclib-treated mice were much smaller than those in vehicle-treated mice (**Figure 6A**). Interestingly, palbociclib exerted stronger inhibitive effects in U87MG-PTEN cells than that in U87MG-Vector cells (**Figure 6A**). Data of the tumor weights were fitted well with the data of the tumor volume (**Figure 6B**). The average tumor weights in drug-treated U87MG-PTEN group were significantly decreased compared to those in drug-treated U87MG-Vector group (**Figure 6B**). These data showed that palbociclib selectively blocked the growth of tumor harboring cells with wild-type PTEN. Furthermore, signaling examinations were carried out with the tumor tissues. Consistent with the *in vitro* data, palbociclib suppressed the levels of phospho-ERK and phospho-Akt in U87MG-PTEN tumors, while no prominent inhibition was observed in tumors bearing U87MG-Vector cells (**Figure 6C**). Quantification data showed that no notable changes of levels of phospho-ERK and phospho-Akt by drug treatment in U87MG-Vector cells. However, signaling of ERK and Akt were significantly suppressed by palbociclib in U87MG-PTEN cells (**Figure 6D**). Hence, these data demonstrate that palbociclib selectively blocks GBM growth *in vivo* through suppression of ERK and Akt signaling.

## Discussion

Heterogeneity is one of the hallmarks of GBM, which causes a variety of phenotypes and therapeutic responses among GBM patients (Gudbergsson et al., 2019; Skaga et al., 2019). Therefore, identification of the biomarkers used for patient classification and targeted drug selection is critical. PTEN, as a key regulator of PI3K/Akt signaling pathway, is demonstrated as a potential prognostic marker of GBM (Kang et al., 2017; Limam et al., 2019). As one of the three key signaling pathways contributing to GBM initiation and progression, PI3K/Akt pathway is a canonical target for GBM treatment (Koul et al., 2006; Narayan et al., 2017). Cyclin D-CDK4/6-Rb signaling pathway is another key signaling pathway conferring GBM, which has not been well developed as target for GBM treatment. Here, with the FDA-approved CDK4/6 inhibitor, palbociclib, we investigated the efficacy of blockade of Rb signaling against GBM and found out the role of PTEN in the potential of clinically applicable anti-CDK4/6 therapies.

Our works revealed that palbociclib selectively suppressed PTEN-expressed human GBM cells *in vitro* and *in vivo*, which was independent on Rb signaling suppression. It has reported that RB pathway alteration strongly correlates with sensitivity to pharmacological inhibition of CDK4/6 in GBM cell lines (Wiedemeyer et al., 2010). The cell lines, LN229, U87MG, LNZ308, U251 and SF763 employed in the present study are all with wild-type RB1 and hyperphosphorylated RB signaling due to dysregulated levels of CDKN2A/2B/2C or/and CDK4/6 (Wiedemeyer et al., 2010). Here, our mechanistic studies showed that the action of PTEN conferring the selectivity of palbociclib in GBM is due to blockade of ERK and Akt signaling (**Figure 6E**). Expression of constitutive active Akt or/and ERK could alleviate or abolish the inhibitive effects of palbociclib in GBM, confirming the relationship between PTEN and palbociclib-induced regulation of ERK and Akt signaling. MAPK/ERK pathway is a critical pathway regulating glioma initiation and development *via* signaling moderators such as receptor tyrosine kinases (RTKs), RAS, etc. (Pandey et al., 2016). Various stimuli such as growth factors, oxidative stress, cytokines, and ischemic injury, can induce its activation. It integrates extracellular and intercellular signals, which is critical for cell proliferation, differentiation, and survival. The PI3K/Akt signaling is triggered by growth factor–receptor interactions. Following activation, PI3K is recruit the cell membrane, leading to the creation of the secondary messenger phosphatidylinositol (3,4,5)-trisphosphate (PIP3) (Oh et al., 2014). Akt is the key effector of PIP3, regulating cell proliferation and survival. As a negative regulator, PTEN can shut down the PIP3 signal, acting as a tumor suppressor (Koul, 2008).

Targeting ERK and Akt signaling pathways for GBM treatment has been extensively studies around the world (Qi et al., 2013a; Ishida et al., 2018). MEK inhibitor, PD0325901, is an effective drug against GBM (Shannon et al., 2017). Most recently, a third-generation EGFR inhibitor AZD9291 exerting anti-GBM activity through blocking ERK signaling has been reported (Liu et al., 2019). For PI3K/Akt inhibitors, more than 50 small molecules have been developed for cancer treatment. However, only small proportion them such as BKM120, XL147, and XL765 have been studied in the clinical trials against GBM (Zhao et al., 2017). BKM120, a pan-PI3K inhibitor, is reported for its potent activity in GBM blockade (Koul et al., 2012). Targeting Rb signaling for GBM treatment is mainly through CDK4/6 inhibitor, which have shown a promising prospect in clinical application (Michaud et al., 2010; Olmez et al., 2017; Qi et al., 2017). However, not all subtypes of GBM respond well to the CDK4/6 inhibitors (Li et al., 2017a), suggesting a patient selection/classification is necessary for targeted treatment.

Both ERK and Akt signaling pathways are downstream of RTK signaling, such as the epidermal growth factor receptor (EGFR), the vascular endothelial growth factor receptor (VEGFR), the platelet-derived growth factor receptor (PDGFR), and the hepatocyte growth factor receptor (HGFR/c-MET) (Qi et al., 2012; Tuncel and Kalkan, 2018). Among them, interaction between PTEN and c-Met signaling has been revealed and PTEN may downregulate ERK signaling through c-Met-dependent signaling pathway (Wang et al., 2000). Indeed, PTEN presence led to the downregulation of p-ERK (**Figures 4A** and **6C**). It is also reported that PTEN deficiency induces glioblastoma malignancy *via* activate c-Met signaling (Li et al., 2009). Our data show that enhanced ERK activity induced by transfection of constitutive active ERK abolished the anti-GBM activity of palbociclib (**Figure 6B**), which implies a potential connection between PTEN and CDK4/6 inhibition. Most recently, Olmez et al. reported that both c-Met and TrkA-B pathways were activated upon CDK4/6 inhibition-induced activation of NF-κB in GBM cells (Olmez et al., 2018). As for the connection between c-Met and CDK4/6, the “OncoPPi network” can be employed for further analysis. It has provided evidence that LKB1 deficient cancer cells were sensitive to CDK4/6 inhibitor based on the protein-protein interaction of LKB1/CDK4, supporting the kinase inhibitors function not only as kinase suppressor, but also as signaling regulator based on protein–protein interaction network (Li et al., 2017b). Indeed, C-Met and cyclin D2, functional partner of CDK4/6, interaction has been revealed (Li et al., 2017b), which indicated the possible regulations among CDK4/6 inhibitor and C-Met. Therefore, probably, in GBM cells, PTEN expression may repress the enhancement of c-Met signaling, which fulfills the efficacy of CDK4/6 inhibitor against GBM. Experiments related these speculations will be carried out in our future studies. To provide further evidence for the application of CDK4/6 inhibitor in GBM treatment, efficacy of a combination of palbociclib and c-Met inhibitor, such as volitinib (Gavine et al., 2015) will be focused, which will also offer the foundation that with c-Met inhibitor, CDK4/6 inhibitor can also be applied for treating GBM with PTEN deficiency.

Taken together, our study demonstrates that CDK4/6 inhibitors selectively suppress PTEN expressed human GBM cells through down-regulation of ERK and Akt signaling, offering a proof-of-concept that suppression two key signaling pathways is a potential strategy for GBM treatment. The findings not only provide insight for the mechanisms by which palbociclib selective suppresses GBM cells with wild-type PTEN, but also offer a foundation for clinical utilization of palbociclib as a potential targeted drug for GBM treatment.

## Data Availability Statement

The datasets generated for this study are available on request to the corresponding author.

## Ethics Statement

The animal study was reviewed and approved by The Committee on the Ethics of Animal Experiments of Jinan University.

## Author Contributions

SL, DY, QQ, BG, SY, LX, TC, and CY conducted the experiments and contributed to data interpretation; SL, DY, JL, BL, LY, and WJ participated in data analysis, discussion, and manuscript preparation. SL, QQ, DY, and WJ designed the experiments and wrote the paper. All authors were involved in the manuscript editing.

## Conflict of Interest

The authors declare that the research was conducted in the absence of any commercial or financial relationships that could be construed as a potential conflict of interest.

## Abbreviations

ATCC, American Type Culture Collection; CDK, cyclin dependent kinase; DMSO, dimethyl sulfoxide; EGFR, epidermal growth factor receptor; GBM, glioblastoma; HGFR, hepatocyte growth factor receptor; MTT, 3-(4,5-dimethylthiazol-2-yl)-2,5-diphenyltetrazolium bromide; PDGFR, platelet derived growth factor receptor; PIP2, phosphatidylinositol 4,5-bisphosphate; PIP3, phosphatidylinositol 3,4,5-trisphosphate; PTEN, phosphatase and tension homolog; Rb, retinoblastoma; RTK, receptor tyrosine kinase; TCGA, Cancer Genome Atlas Research Network; VEGFR, vascular endothelial growth factor receptor.

## Supplementary Material

Supplemental Figure 1 Representative images of the effects of palbociclib on colony formation in different GBM cell lines.

Supplemental Figure 2 PTEN modulates the sensitivity of GBM cells to palbociclib. **(A)** PTEN knocking-down decreased the sensitivity of SF763 cells to palbociclib. Following PTEN/control siRNA transfection, cells were treated with palbociclib at indicated concentrations for 48 hours; cell proliferation was examined by MTT assay. Data are expressed as mean ± SD. **(B)**. Expression of PTEN enhanced the efficacy of palbociclib against U251 cells. Following PTEN transfection, U251 cells were treated with palbociclib at indicated concentrations for 48 hours, cell proliferation was assessed by MTT assay. Data are expressed as mean ± SD.

Supplemental Figure 3 PTEN modulates the sensitivity of GBM cells to palbociclib. Cells were prepared as described in **Figure 5A** and treated with palbociclib at different concentrations. Cell proliferation was assessed by BrdU incorporation assay. Data were expressed as mean ± SD (*P < 0.05, **P < 0.01, vs. control cells in each dose group, t-test, n = 3).
