# Supplementary figures and images for: Involvement of Phosphatase and Tensin Homolog in Cyclin-Dependent Kinase 4/6 Inhibitor-Induced Blockade of Glioblastoma
Source: Front Pharmacol. 2019 Nov 7;10:1316. doi: 10.3389/fphar.2019.01316 (PMC6854038; doi:10.3389/fphar.2019.01316)

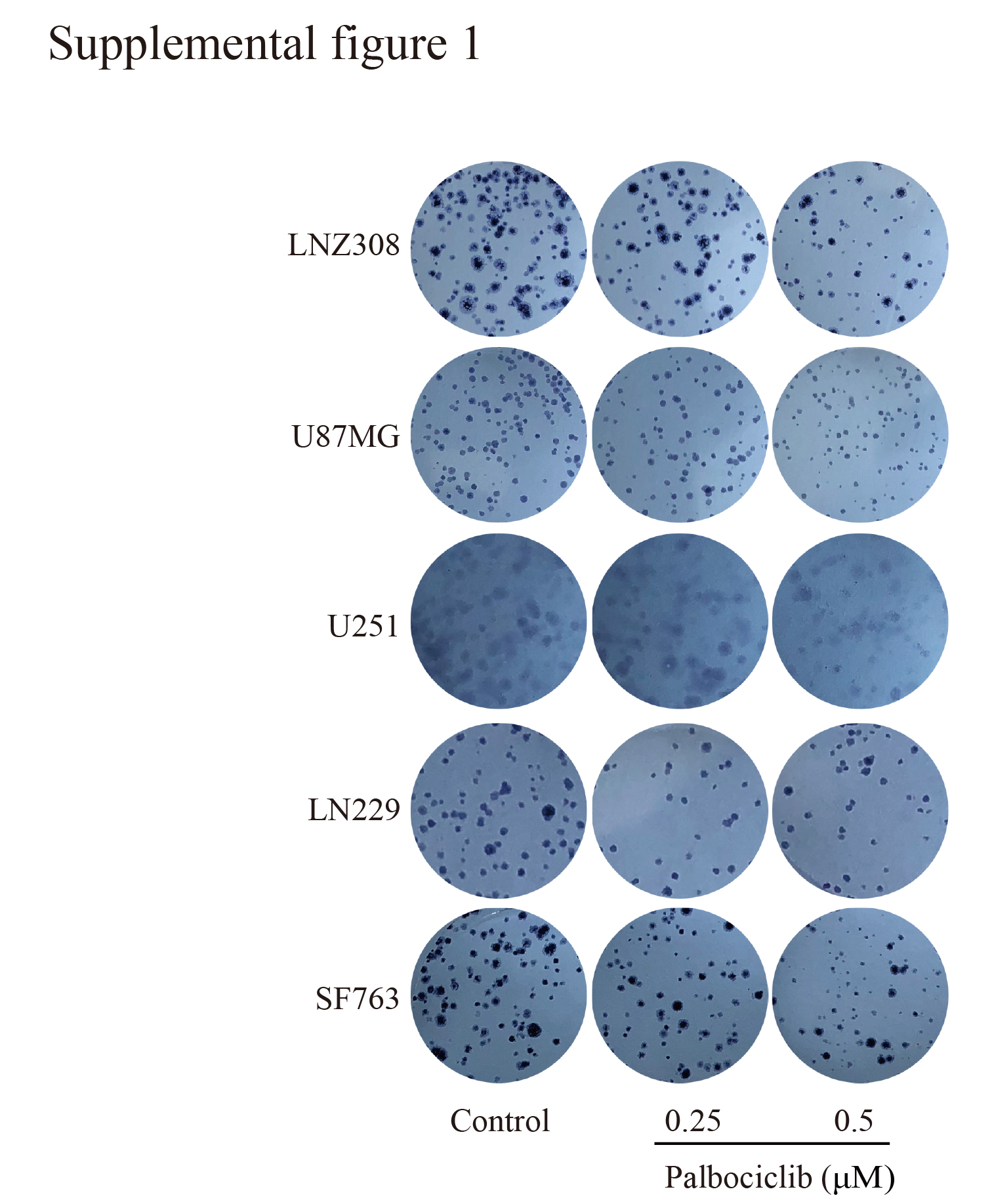

Supplement: Supplemental Figure 1 — Representative images of the effects of palbociclib on colony formation in different GBM cell lines. [file Image_1.jpeg]

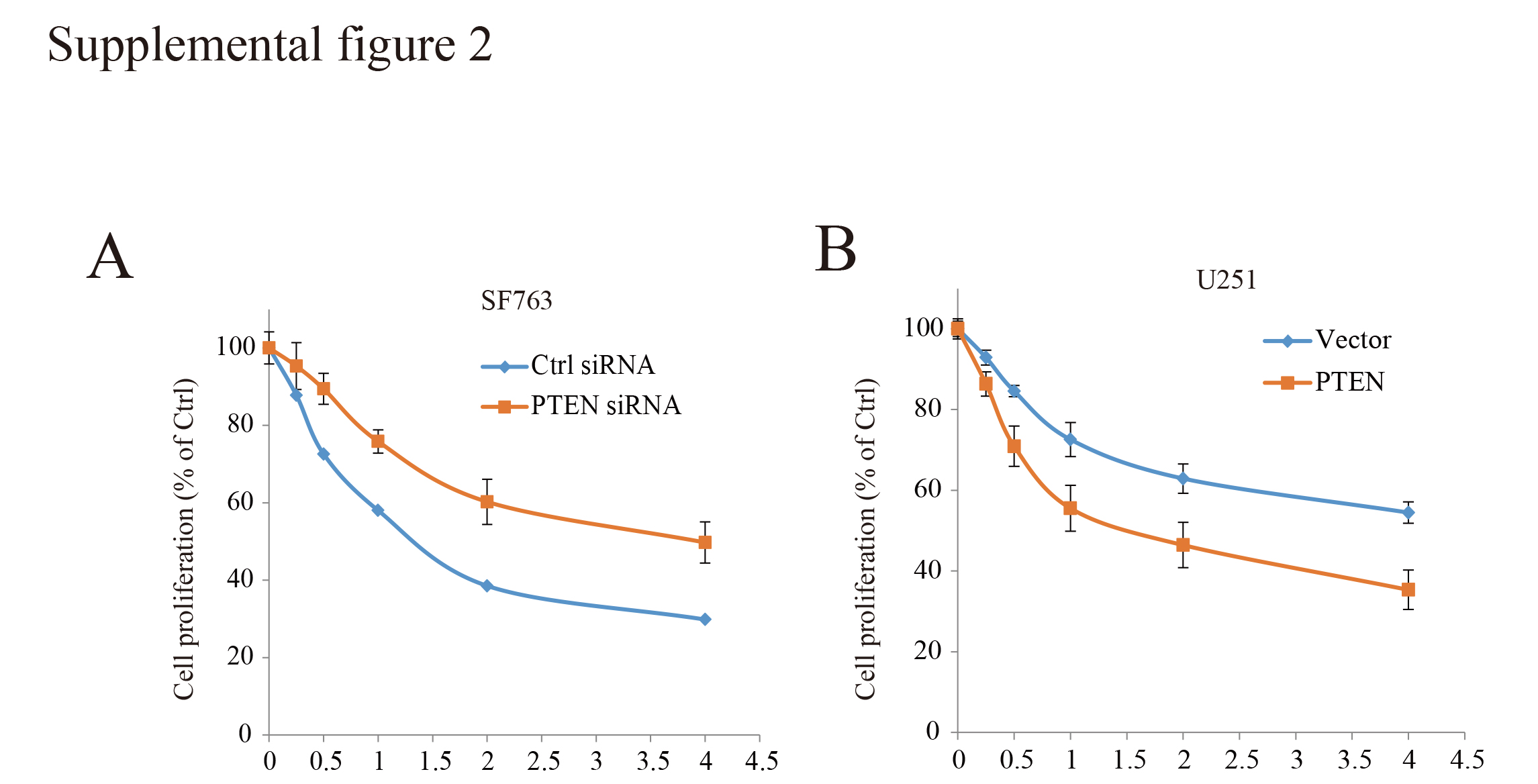

Supplement: Supplemental Figure 2 — PTEN modulates the sensitivity of GBM cells to palbociclib. (A) PTEN knocking-down decreased the sensitivity of SF763 cells to palbociclib. Following PTEN/control siRNA transfection, cells were treated with palbociclib at indicated concentrations for 48 hours; cell proliferation was examined by MTT assay. Data are expressed as mean ± SD. (B). Expression of PTEN enhanced the efficacy of palbociclib against U251 cells. Following PTEN transfection, U251 cells were treated with palbociclib at indicated concentrations for 48 hours, cell proliferation was assessed by MTT assay. Data are expressed as mean ± SD. [file Image_2.jpeg]

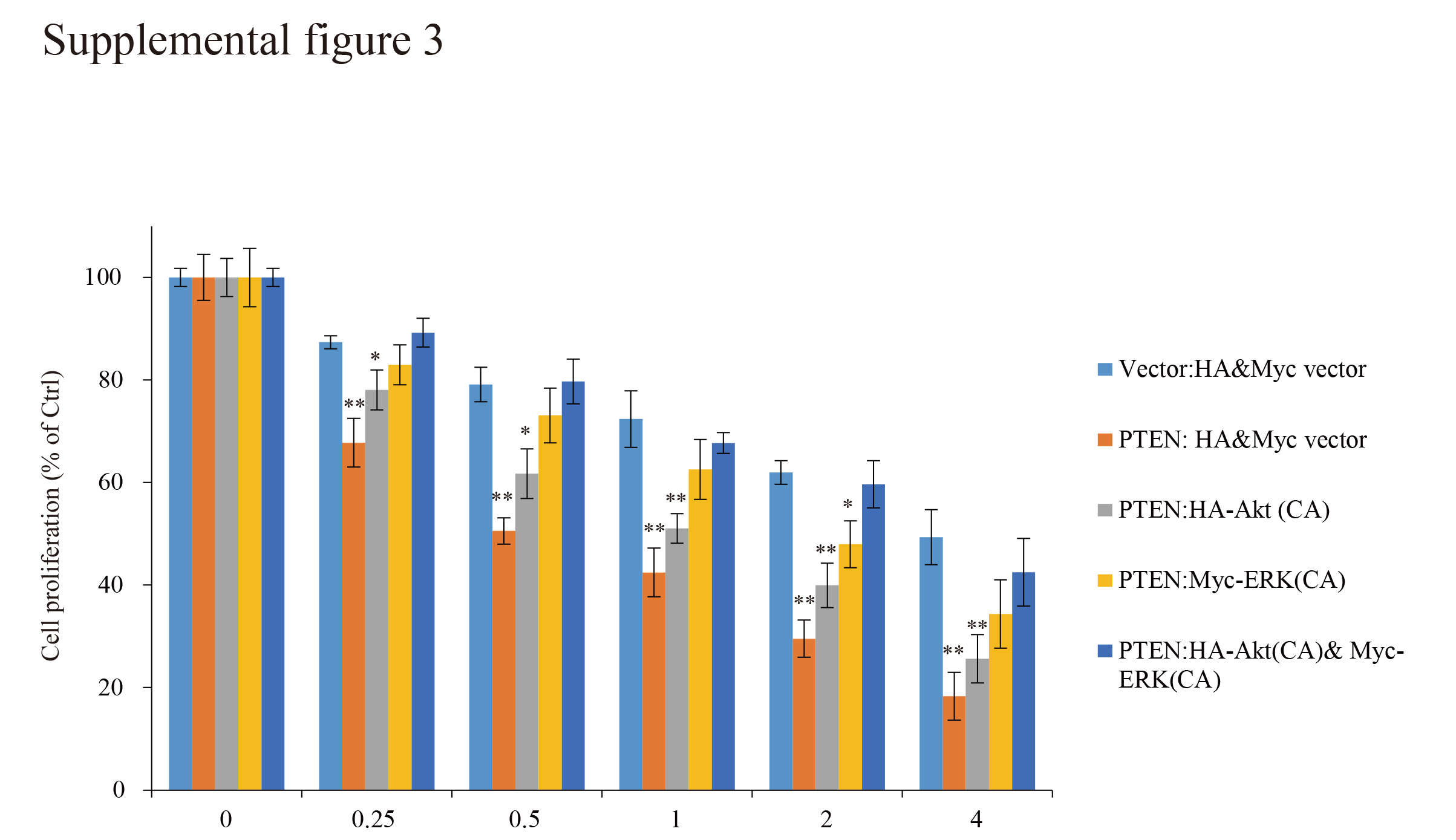

Supplement: Supplemental Figure 3 — PTEN modulates the sensitivity of GBM cells to palbociclib. Cells were prepared as described in Figure 5A and treated with palbociclib at different concentrations. Cell proliferation was assessed by BrdU incorporation assay. Data were expressed as mean ± SD (*P < 0.05, **P < 0.01, vs. control cells in each dose group, t-test, n = 3). [file Image_3.jpeg]
